# Mitochondria Transfer to CD4^+^ T Cells May Alleviate Rheumatoid Arthritis by Suppressing Pro-Inflammatory Cytokine Production

**DOI:** 10.20900/immunometab20220009

**Published:** 2022-03-18

**Authors:** Rocky Giwa, Jonathan R. Brestoff

**Affiliations:** Department of Pathology and Immunology, Washington University School of Medicine, St. Louis, MO 63110, USA

**Keywords:** mitochondria, CD4 T cells, rheumatoid arthritis

## Abstract

CD4^+^ T cells contribute to the pathogenesis of autoimmune diseases such as rheumatoid arthritis (RA). These cells infiltrate the joints of RA patients and produce cytokines, including Tumor necrosis factor (TNF)-α, that drive joint inflammation and bone destruction. Although biologic therapeutics targeting T cells and TNF-α have benefited patients suffering from RA, some patients are refractory to these therapies, develop antibodies that neutralize these biologics, or develop undesirable side effects. Recent studies indicate that CD4^+^ T cell cytokine production is regulated in part by specific metabolic modules, suggesting that immunometabolic pathways could represent a novel therapeutic strategy for T cell-mediated diseases such as RA. Wu et al. (2021) demonstrate that mitochondrial function is impaired in CD4^+^ T cells from RA patients, leading to reduced levels of various citric acid cycle metabolites (e.g., aspartate) that regulate TNF-α production. Treatment of RA-associated T cells with purified mitochondria was sufficient to restore these metabolic defects, limit production of numerous pro-inflammatory cytokines such as TNF-α and IL-17A, and reduce the development of RA-like disease in a humanized mouse model. These data suggest that T cells can be metabolically “re-engineered” ex vivo with exogenous mitochondria and that this mitochondria transfer approach confers anti-inflammatory properties that may reduce disease severity in RA and possibly other rheumatologic diseases. Increasing our understanding of how intercellular mitochondria transfer occurs may identify novel biological pathways that can be targeted therapeutically or harnessed to support cell engineering.

T cells are responsible for coordinating antigen-specific immune responses for host defense against pathogens but can also contribute to chronic inflammatory conditions, anti-tumor immunity, and autoimmune disease pathogenesis [1]. Rheumatoid arthritis (RA) is an autoimmune disease characterized by joint inflammation, joint pain and bone destruction. Although multiple studies have revealed that proinflammatory cytokines (e.g., Tumor necrosis factor [TNF]-α and Interleukin [IL]-1β), metabolites (e.g., arachidonic acid), and other factors (e.g., autoantibodies) contribute to the intertwined innate and adaptive immune responses seen in RA, the mechanisms underlying this complex disorder remain elusive [2]. In RA, the synovium is infiltrated by T cells, monocytes, B cells, and other cell types that collectively produce proinflammatory cytokines that contribute to joint pain and damage [3]. In particular, T cells are the most abundant immune cell population in the leukocyte-rich synovial fluid from RA-afflicted joints and appear to be the dominant source of TNF-α locally [4]. The success of therapeutics for RA that target either T cells or TNF-α strongly implicates these factors in this disease in humans, however some RA patients are refractory to these treatments, develop antibodies that neutralize these biologics, or experience various side effects that can range from mild (e.g., rash) to severe (e.g., sepsis) [5,6]. Therefore, it is essential that we increase our understanding of the cellular and molecular mechanisms that regulate RA-associated inflammation, as this could lead to the identification of novel therapeutic strategies for RA.

It has become clear that metabolic pathways directly regulate T cell function. For example, it has been shown that CD4^+^ Th1 cell production of Interferon (IFN)-γ is regulated in part at the post-transcriptional level [7]. In resting Th1 cells, the glycolytic enzyme Glyceraldehyde-3-phosphate dehydrogenase (GAPDH) binds the fully processed mRNA of *Ifng*, thereby inhibiting its translation. However, upon stimulation of the TCR, aerobic glycolysis (i.e., Warburg metabolism) is induced, leading to dissociation of GAPDH and the *Ifng* transcript. This step allows GAPDH to participate in aerobic glycolysis while simultaneously releasing the *Ifng* mRNA to be quickly translated into protein and secreted. As another example, after resolution of an adaptive immune response, the formation and maintenance of memory CD8^+^ T cells involves a switch in energy substrate utilization from glucose to fatty acids [8]. While there are several other examples of metabolic regulation of T cells [9], these studies highlight that cellular metabolic pathways can regulate specific functional modules in T cells. However, the metabolic factors that regulate T cells and their production of TNF-α in autoimmune diseases such as RA are not fully understood.

Previous studies have demonstrated that naïve T cells from patients with RA exhibit decreased adenosine triphosphate (ATP) concentrations and oxidative stress due to a disruption in glucose flux, resulting in increased proliferation and migration of CD4^+^ T helper type 1 (Th1) cells [10,11]. These changes in RA-associated T cells suggest there may be immunometabolic mechanisms underlying the pathogenesis of this autoimmune disease. In a recent study, Wu et al. (2021) expand our understanding of immunometabolic regulation of T cells in RA by identifying a key role for the mitochondrial metabolite aspartate in regulating TNF-α production (Figure 1A) [12]. To begin, the authors found that mitochondria in naïve and unstimulated T cells obtained from RA patients have impaired mitochondrial membrane potential and aerobic respiration compared to T cells from age-matched healthy individuals. Experimental perturbation of aerobic respiration in healthy T cells using rotenone, an electron transport chain complex I inhibitor, was sufficient to induce an increase in endoplasmic reticulum (ER) biomass, suggesting a potential link between mitochondrial metabolism and ER morphology. Screening of citric acid cycle intermediates following rotenone treatment indicated that there was reduced aspartate levels, and this metabolic change was associated with increased ER biomass and induced expression of TNF-α in healthy human T cells. Conversely, aspartate supplementation reduced T cell ER biomass and TNF-α expression in human T cells, and administering aspartate to mice with RA-like disease ameliorated joint pathology and proinflammatory cytokine expression, including *Tnf*. These studies suggest that mitochondria-derived metabolites, such as aspartate, can modulate T cell-mediated inflammation in RA (Figure 1B).

In an attempt to rescue mitochondrial metabolism in RA-associated T cells, the authors exposed CD4^+^ T cells from RA patients to purified mitochondria isolated from either healthy or RA CD4^+^ T cells. Recent studies have shown that various cell types, including ρ^0^ cells that lack fully functional mitochondria, can capture purified mitochondria from their environment to support their metabolic demands [13,14]. Remarkably, Wu et al. (2021) [12] found that mitochondria treatment of healthy but not RA CD4^+^ T cell-derived mitochondria, increased aspartate concentrations and the NAD^+^:NADH ratio in T cells, and this was associated with not only reduced ER biomass but also decreased TNF-α expression. Furthermore, while adoptive transfer of RA-associated CD4^+^ T cells from patients led to RA-like joint pathology and inflammation in humanized chimeric NSG mice, pre-treating these T cells with exogenous healthy mitochondria substantially ameliorated the development of joint disease (Figure 1C). These data suggest that treating T cells with exogenous mitochondria ex vivo may hold promise as a therapeutic strategy against RA by conferring an anti-inflammatory phenotype. The transferred mitochondria may modulate this anti-inflammatory phenotype by restoring aerobic respiration in T cells or by delivering intermediary metabolites such as aspartate, as both scenarios would be expected to replenish aspartate levels in recipient cells. As shown by Wu et al. (2021) [12], aspartate then rapidly suppresses ER expansion in RA T cells and thereby limits production of TNF-α and RA pathogenesis. The authors also observed that decreased concentrations of NAD^+^, a cytosolic metabolite downstream of aspartate metabolism, impairs the ability of CD4^+^ T cells to activate an ER stress pathway controlled by the chaperone GRP78/BiP. Administering NAD^+^ to CD4^+^ T cells was sufficient to restore this BiP-dependent ER stress response pathway, resulting in reduced ER biomass and less severe RA-associated inflammation.

Several other recent studies support the observation that T cells can receive mitochondria from other cell types and suggest that this process may affect T cell development and/or function. For example, one study demonstrated that T cells can obtain mitochondria from mesenchymal stem cells (MSCs) and that this process promotes regulatory T (T_reg_) cell differentiation and production of IL-10 [15]. IL-10 is an anti-inflammatory cytokine that dampens inflammation and is required to prevent the development of autoimmune diseases [16]. Similarly, it was reported that MSCs can transfer mitochondria to CD4^+^ T helper type 17 (Th17) cells and that this process increases aerobic respiration of T cells, reduces expression of IL-17A, and may promote interconversion of Th17 cells into T_reg_ cells [17]. This MSC-to-Th17 mitochondria transfer axis is impaired when the MSCs are derived from RA patients. Although Wu et al. (2021) [12] did not find that IL-10 expression was altered in T cells following aspartate treatment or mitochondria transfer, they did report reduced IL-17A expression in joint tissue from mice with RA-like disease. It is not yet clear whether mitochondria transfer to T cells is impaired in RA in vivo or whether this process contributes to RA pathogenesis.

There are several open questions regarding mitochondria transfer to T cells. First, it is not clear whether mitochondria transfer to T cells occurs appreciably in vivo or which factors regulate this process. Second, it has not been shown that any human cells can transfer mitochondria to each other in vivo, mainly due to technical limitations and an inability to measure mitochondria transfer in humans. Although Wu et al. (2021) [12] used humanized NSG chimeric mice, where their immune system is replenished with human immune cells, they did not directly measure or observe mitochondria transfer in vivo. Third, it is not clear how T cells are able to capture exogenous mitochondria. CD4^+^ T cells have low phagocytic efficiency compared to macrophages, the latter of which we and others demonstrated frequently obtain mitochondria from neighboring cells in their local tissue microenvironment [18,19]. Recent work indicates that macrophages can export mitochondria to nearby cells, at least in the dorsal root ganglia [20]. Since macrophages and CD4^+^ T cells frequently interact, it is possible that macrophages may first acquire mitochondria from parenchymal or stromal cells in the joint (or other tissues) and then subsequently deliver those mitochondria to CD4^+^ T cells.

Overall, this study by Wu et al. (2021) [12] contributes exciting new knowledge to our understanding of autoimmune disease pathogenesis. The authors describe a novel mechanism by which mitochondrial metabolism in T cells regulates ER morphology and pro-inflammatory cytokine production in RA. The data suggest several possible new therapeutic approaches to RA. One is supplementation with intermediary metabolites of the citric acid cycle, such as aspartate, to suppress the inflammatory T cell response in the joint. Alternatively, it may be possible to isolate T cells from RA synovial fluid and modify them ex vivo with exogenous healthy mitochondria to inhibit proinflammatory cytokine production, followed by reinfusion into the joint. A recent study showed that exogenous mitochondria can enhance engraftment of bone marrow, suggesting that ex vivo mitochondria transfer could be harnessed in multiple therapeutic contexts [21]. At least three startup biotech companies (Minovia, Mitrix, and Cellvie) are attempting to develop the use of exogenous mitochondria for therapeutic purposes. The study by Wu et al. (2021) [12] suggests that autoimmune diseases such as RA may represent an attractive category of diseases to treat with this approach.

## Figures and Tables

**Figure 1.**
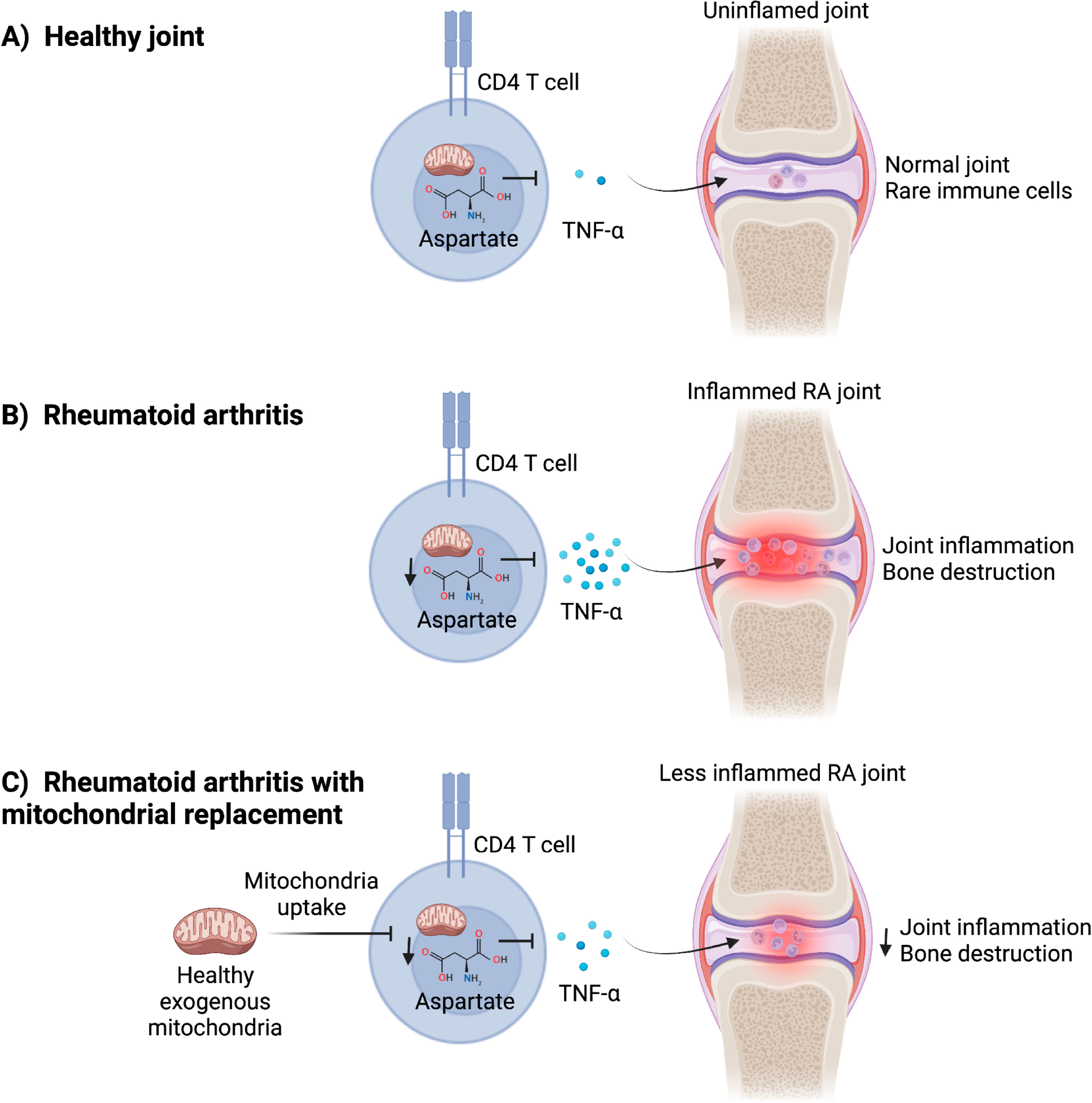
Exogenous mitochondria restore aspartate levels in CD4^+^ T cells to limit production of TNF-α and joint inflammation in rheumatoid arthritis-like disease. (**A**) In the healthy joint, CD4^+^ T cells are rare and have normal mitochondrial metabolism that allows adequate concentrations of citric acid cycle intermediary metabolites, such as aspartate, to be produced. Aspartate suppresses production of proinflammatory cytokines, such as Tumor necrosis factor (TNF)-α. (**B**) CD4^+^ T cells from rheumatoid arthritis (RA) patients have reduced mitochondrial function and decreased concentrations of aspartate. The inhibitory effect of aspartate on TNF-α production is removed, leading to upregulated expression of this cytokine to drive inflammation and bone destruction in RA-afflicted joints. (**C**) Treating RA patient-derived T cells with healthy exogenous mitochondria restores mitochondrial metabolism in T cells, increases aspartate concentrations, and inhibits expression of TNF-α and other pro-inflammatory cytokines. Ex vivo mitochondria treatment of RA T cells attenuated joint inflammation and bone destruction in a humanized mouse model of RA-like disease, suggesting that mitochondria transfer could be utilized to “metabolically engineer” T cells for the treatment of rheumatologic diseases, such as RA.
